# Regulatory role of the intestinal microbiota in the immune response against *Giardia*

**DOI:** 10.1038/s41598-021-90261-z

**Published:** 2021-05-19

**Authors:** B. Maertens, A. Gagnaire, O. Paerewijck, K. De Bosscher, P. Geldhof

**Affiliations:** 1 Department of Virology, Parasitology and Immunology, Laboratory of Parasitology, Faculty of Veterinary Medicine, Ghent University, Merelbeke, Belgium; 2 VIB Department of Medical Protein Research, Translational Nuclear Receptor Research Lab, Faculty of Medicine and Health Sciences, Ghent University, Ghent, Belgium

**Keywords:** Infection, Parasite host response

## Abstract

*Giardia duodenalis* is one of the most commonly found intestinal parasites in mammalian hosts. Infections can generally be cleared by mounting an adequate protective immune response that is orchestrated through IL-17A. This study was aimed to investigate if and how the intestinal microbiome affects the protective Th17 response against *Giardia* by analysing and comparing the immune response following a *G. muris* and *G. duodenalis* infection in antibiotic treated and untreated mice. Depletion of the intestinal flora by antibiotic treatment had a severe effect on the infection dynamics of both *Giardia* species. Not only duration of infection was affected, but also the parasite burden increased significantly. Markers associated with a protective immune response, such as IL-17A and mannose binding lectin 2 were still significantly upregulated following infection in the antibiotic-treated mice, despite the lack of protection. On the other hand, the antibiotic treatment significantly decreased the level of IgA in the intestinal lumen by affecting its transporter and by reducing the number of IgA^+^ B-cells at the Peyer’s patches. Furthermore, the depletion of the gut microbiota by antibiotics also significantly lowered the intestinal motility. The combination of these factors likely results in a decreased clearance of the parasite from the intestinal tract.

## Introduction

With more than 200 million cases a year worldwide, *Giardia duodenalis* is one of the most common intestinal parasites in humans, especially in developing countries^1^. *Giardia* infects both children as adults and causes the enteric infection giardiasis, in which fatty diarrhea, nausea and flatulence are common symptoms^2^. However, many infected individuals do not show any symptoms and function as carriers, suggesting that the true prevalence of *Giardia* infections is even higher than estimated^3^. Infections occur through the fecal–oral route by ingestion of contaminated food or drinking water. Recent studies have highlighted the importance of interleukin 17A (IL-17A) as the key factor in the development of intestinal immunity against *Giardia*^4,5^. Both *G. muris* and *G. duodenalis* infection studies in mice showed a significant upregulation of IL-17A starting one week after infection. Furthermore, it was shown that mice deficient in the IL-17A receptor or IL-17 itself lose their ability to clear the infection^4,5^. The most important downstream effectors of the IL-17A response, that have been shown to play a critical role in the clearance of a *Giardia* infection, are the activation of the complement system by mannose-binding lectin 2 (MBL2) and the production and secretion of parasite-specific IgA’s^4,6,7^. CD4 + T cells are one of the important cellular sources of IL-17A following a *Giardia* infection^4^. The differentiation of these Th17 cells is driven by IL-6 and the retinoic acid-related orphan receptor RORgT, which are both upregulated in an early phase of a *Giardia* infection^5,8^.

In steady state, Th17 cells are typically abundant in the lamina propria of the small intestine^9^. However the amount of Th17 cells in the lamina propria are extremely reduced in germfree mice and mice treated for four weeks with an antibiotic cocktail of neomycin, ampicillin, vancomycin and metronidazole, indicating that the intestinal microbiome is important for the differentiation of Th17 cells in the small intestine^10^. Furthermore, in mammalian neonates, it has been shown that the microbial colonization of the gut is necessary for the development and education of a functioning immune system^11,12^. These observations suggest that the host intestinal microbiome might also play an important role in the induction of the protective IL-17A response following a *Giardia* infection. Neonatal mice for example, infected with *G. muris,* display a prolonged course of infection and a delayed IL-17A response compared to older mice^13^. Furthermore, the oral administration of antibiotics makes mice more susceptible to a *G. duodenalis* infection and results in a higher parasite burden compared to untreated mice^14^.

In order to gather more insights in the role of the microbiome to mount an anti-*Giardia* immunity, we analysed and compared the intestinal immune responses following a *G. muris* infection in antibiotic treated versus untreated mice and we set out to unravel the mechanism by which antibiotic-treated mice are more susceptible to a *G. duodenalis* infection.

## Material and methods

### Ethical statement

All animal experiments were conducted in accordance with the European Union (E.U.) Animal Welfare Directives, the VICH Guidelines for good Clinical Practice and in compliance with the ARRIVE guidelines (https://arriveguidelines.org). Ethical approval to conduct the studies was obtained from the Ethical committee of the Faculty of Veterinary Medicine, Ghent University (EC2019/10).

### Murine infection studies

All infection studies were performed in female C75 BL/6 mice (Charles River) of 6 weeks old at the time of infection. Before the start of the study, mice were co-housed in groups of 5 animals. Following antibiotic treatment and/or infection, mice were transferred to smaller cages and co-housed with 2 or 3 animals. The antibiotic treatment was administered through the drinking water and consisted of 1.4 mg/ml neomycin, 1 mg/ml ampicillin or 1 mg/ml vancomycin or a combination of all three. Treatment was started five days prior to infection and was kept until the end of the experiment. Mice were infected with either 10^3^ *G. muris* cysts suspended in 0.1 ml phosphate-buffered saline (PBS) or 1 million *G. duodenalis* (assemblage B—GS/M strain) tropohozoites both by oral gavage. In order to monitor the course of the infection, faecal cyst counts were performed at regular time points (indicated in the Figure legends) following infection. Cysts were isolated from faecal pellets by a centrifugation step at low speed over a 1 M sucrose gradient and microscopically counted with a hemacytometer as previously described by Roberts-Thomson and Mitchell^15^. Animals were euthanized at different timepoints following infection (day 7, 14 and 21) by cervical dislocation. The fifth cm of the small intestine was isolated and snap-frozen in liquid nitrogen and stored at − 80 °C for further analysis. The remainder of the small intestine was opened longitudinally and incubated in ice-cold PBS for 20 min in order to count the number of *Giardia* trophozoites. Subsequently, when necessary the solution was diluted, followed by counting the number of trophozoites with the use of a hemacytometer.

### Bacterial load

16S rRNA levels were determined in both feces and small intestinal content. For feces, samples were collect at all timepoints, i.e. before the start of the antibiotic treatment (day − 5), at the day of infection (day 0) and at day 14 and 21 post *G. muris* infection. Intestinal content samples were collected at necropsy at day 21 post *G. muris* infection. Both samples were immediately snap-frozen and stored at − 80°. DNA extraction was performed on 0.025 g feces or 300 µl intestinal content using the QIAamp DNA stool mini kit (Qiagen) according to manufacturer’s instruction with the addition of a 5 min heating step at 95 °C during homogenisation. The quantitative PCR was performed using the StepOnePlus real-time PCR system (Applied Biosciences) under the following conditions: 95 °C for 10 min; 40 cycles with 1 cycle of denaturation at 95 °C for 30 s and 1 cycle for both annealing and extension at 60 °C for 1 min.

### RNA isolation and qRT-PCR

For intestinal gene expression analysis, a 1 cm long fragment of the duodenum was taken at 4 cm from the gastroduodenal junction and snap-frozen in liquid nitrogen. The samples were next disrupted and homogenised using a Tissuelyser II (Qiagen). RNA extraction was subsequently performed using the RNeasy minikit (Qiagen) according to the manufacturer’s instructions. Removal of genomic DNA was done by an on-column RNase-free DNase set (Qiagen). Total RNA concentrations were measured using a Nanodrop ND-1000 spectrophotometer (Nanodrop Technologies) and the quality was verified by an Experion automated electrophoresis system (Bio-Rad). To obtain cDNA for qRT-PCR analysis, the iScript cDNA synthesis kit was used (Biorad).

All qRT-PCR analyses were performed using the StepOnePlus real-time PCR system (Applied Biosciences) under the following conditions: 95 °C for 20 s; 35 cycles with 1 cycle of denaturation at 95 °C for 5 s and 1 cycle for both annealing and extension at 60 °C (the optimal annealing temperature for all the genes examined). For all reactions, SYBR green master mix (Applied Biosystems) was used with 10 ng of single-stranded cDNA and 500 nM of primer. Primer sets for every gene were designed using Primer3 software in a exon-exon spanning manner in order to control for potential genomic DNA contamination (see Appendix for sequences). All samples were analysed in duplo and a non-template control was included in each assay. Melting curve analyses were performed post-analysis to ensure the specificity of the primers. Relative quantities were calculated out of the threshold cycle value using the ΔC_T_-method (Q = E^min Ct − sample Ct^; Q represents the sample quantity relative to the sample with the highest transcription; E represents amplification efficiency, which was measured based on a standard dilution curve obtained by serial dilutions of pooled cDNA material from all samples; min Ct equals the lowest C_T_ value). The obtained values were normalized in gNORm with the housekeeping genes TATA box binding protein (TBP) and Hypoxanthine phosphoribosyltransferase 1 (HPRT1) as previously described^5^.

### Flow cytometry

Peyer’s patches (PP) were isolated from the small intestine and cells were extracted as previously described^16^. Briefly, PP were finely cut and incubated for 40 min at room temperature with collagenase/DNase. Cells were then filtered through a 70 µm cell strainer and pelleted by centrifuging at 300×*g*, 4 °C during 5 min and then resuspended in FACS buffer (PBS, 2% FCS, 5 mM EDTA) and counted before antibody staining. For flow cytometry, cells were first incubated on ice in FACS buffer containing an anti-CD16/CD32 antibody to block the Fc receptor for 10 min and then incubated 30 min on ice in the dark with antibodies against the following surface markers : CD45-PE-eF610 (eBiosciences, clone 30-F11), CD19-PerCp-Cy5.5 (Biolegend, clone 6D5), CD3-PeCy7 (eBiosciences, clone 145-2C11), B220-AF647 (BD Biosciences, clone RA3-6B2), IgD-BV421 (BD Biosciences, clone 11-26c.2a), IgM-FITC (BD Bioscience, clone II/41) and IgA-PE (eBiosciences, clone mA-6E1). Cell viability was evaluated using Fixable Viability Dye eFluor 506 (eBiosciences). Cell acquisition was performed using a CytoFlex (Beckman Coulter) and data were analyzed with the CytoExpert software (Beckman Coulter).

### ELISA

Luminal IgA levels from the small intestine were measured by an enzyme-linked immunosorbent assay (ELISA). The small intestine was removed from mice after necropsy and incubated overnight at 4 °C in PBS with 0.01% sodium azide and 1% protease inhibitor cocktail (Sigma-Aldrich). The IgA containing supernatants were transferred to a new tube after centrifugation at 16,000×*g* for 10 min and stored at − 80 °C. For the sandwich ELISA, maxisorb 96-well plates were coated with 2 µg/ml sheep anti-mouse IgA (Sigma-aldrich) for 16 h at 4 °C. After washing with PBS-Tween 20 (0.05%) (PBST), plates were subsequently blocked with 2% bovine serum albumin (BSA) in PBST. Luminal extracts were added to the plates in a 1/10 dilution and incubated for 1 h at room temperature followed by a washing step with PBST. Detection of IgA was performed by adding goat anti-mouse IgA-HRP (Sigma-Aldrich) in a 1/1000 dilution in PBST followed by a visualisation by ABTS in ABTS buffer (Roche). Finally, optical density was measured at 405 nm with a Tecan plate reader moderated by conjugate control levels measured at 492 nm.

### Intestinal motility

To measure the intestinal motility, all mice were starved overnight with water available freely. Mice were orally gavaged with 0.2 ml of 10% charcoal (Sigma-Aldrich) in 5% gum acacia (Sigma-Aldrich) and were sacrificed 20 min after administration. Subsequently, the small intestine was removed and measured from pylorus till the cecum. Next, the distance travelled by the dye was measured from pylorus till the leading edge of the charcoal dye. Motility was set as the percentage of the distance travelled by the dye.

### Statistics

Statistical analysis for gene expression, IgA ELISA and flow cytometry were performed by GraphPad Prism software using non-parametric Kruskal–Wallis test followed by a Dunn’s multiple-comparison test to determine significant differences between both infected and non-infected mice as between antibiotic treated and non-treated groups. Differences were considered significant at a p-value of ≤ 0.05. For trophozoite counts, area under the curve analysis and intestinal motility a Mann–Whitney U test was performed in order to detect significant differences. A p-value ≤ 0.05 was considered significant.

## Results and discussion

### Reduction of the intestinal microbiota enables *G. duodenalis* infection in mice and prolongs the course of a *G. muris* infection

The *G. duodenalis* infection model in adult mice was originally developed to overcome several limitations with existing infection models like *G. muris* in mice and *G. duodenalis* in gerbils^17^. Yet also this model encounters limitations as infection by oral ingestion of cysts requires a high amounts of cysts which are difficult ot produce. For this reason, it is typically replaced by oral gavage of trophozoites which can be grown in vitro^18^. Furthermore, it has been described that *G. duodenalis* infection in mice displays a variable success rate, which could be overcome by oral administration of antibiotics, indicating the importance of the intestinal microbiome for susceptibility. Singer et al.^14^ showed that mice with a conventional microbiome from one supplier were susceptible to infection whereas mice from another supplier were not. Later work showed that a WB strain of *G. duodenalis* required an antibiotic treatment prior to infection whereas the GS strain did not^15^.

**Figure 1:**
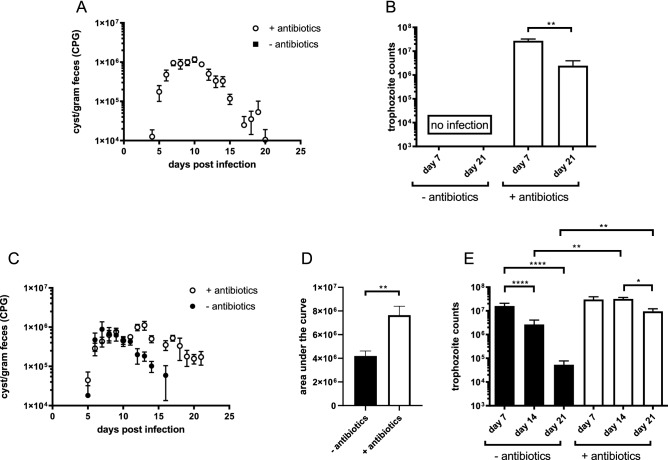
Dynamics of *Giardia* infections in mice following antibiotic treatment. (**A**) *G. duodenalis* cysts present in the feces of antibiotic-treated and untreated mice were monitored daily until day 21 p.i. Mean numbers of cysts per gram faeces obtained from 5 mice at every time point are depicted, with SEM as error bars. (**B**) *G. duodenalis* trophozoite numbers in the small intestine of antibiotic-treated C57Bl/6 mice at indicated time points. No trophozoites were detected in the untreated mice. (**C**) *G. muris* cysts present in the faeces of antibiotic-treated and untreated mice were monitored from day 4 p.i. until day 21 p.i. Mean numbers of cysts per gram faeces obtained from 5 mice at every time point are depicted, with SEM as error bars. (**D**) Area under the curve analysis of cyst counts between antibiotic treated and untreated C57Bl/6 mice infected with *G. muris* (**E**) *G. muris* trophozoite numbers in the small intestine of antibiotic-treated and untreated C57Bl/6 mice at indicated time points. (*p ≤ 0.05, **p ≤ 0.01, ***p ≤ 0.001, ****p ≤ 0.0001).

In this study a *G. duodenalis* infection in C57BL/6 mice with both a conventional microbiome and an antibiotic-depleted microbiome was performed. The results confirmed previously described findings by Singer et al.^14^, that mice with a conventional microbiome indeed did not develop a measurable infection (Fig. 1A) as both parameters indicative of a *Giardia* infection, namely faecal cysts secretion and trophozoites in the duodenum, were absent. However, antibiotic treatment resulted in a marked infection with cyst secretion in the faeces starting around day 5 post infection (p.i.) and with a peak between day 7 and 10 p.i. followed by a decrease towards day 21 p.i. (Fig. 1A). A similar pattern was also seen for the trophozoite counts with a peak early in the infection and a decrease of the parasite burden at day 21 p.i. (Fig. 1B). These findings indicate an important role for the microbiome in the establishment of a *G. duodenalis* infection whereas the ability of the animals to clear the infection remains intact.

In a next phase, the same approach was used to investigate the effect of an antibiotic treatment on a *G. muris* infection, with the inclusion of an additional time point at day 14 p.i. for trophzoites counts in order to compare with previously performed studies^5,7^. Cyst counts (Fig. 1C) of mice without antibiotics displayed a normal pattern with a peak of infection at day 7 p.i., after which the number of cysts in the faeces decreased to the detection limit at day 21p.i. Mice with antibiotic treatment however, showed a delayed peak of cyst secretion at day 13 p.i. followed by a slower decrease of cyst levels. At day 21 p.i., antibiotic treated mice still secreted higher numbers of cysts in their faeces in comparison to untreated mice. Overall, the area under the curve analysis (Fig. 1D) of the cyst counts showed a significant increase in cyst excretion. To monitor the course of infection more closely, trophozoites counts (Fig. 1E) were performed after necropsy at day 7, 14 and 21 p.i. for both groups. A first observation during necropsy was the extreme enlargement of the cecum in all antibiotic treated mice compared to mice with a normal microbiota (Supplementary Data, S1), which has already been described in the past^19^. The number of trophozoites was similar for both groups at day 7 p.i., indicating the onset of infection was not affected by the antibiotic treatment. The untreated mice subsequently showed a steady decrease in the number of trophozoites after day 7 p.i. whereas this was not the case for the antibiotic treated mice. Furthermore, trophozoite counts were both on day 14 and 21 p.i. significantly higher in the antibiotic treated mice compared to the untreated mice. To examine the effect of the antibiotics separately, additional infection studies were performed in which neomycin, ampicillin and vancomycin were tested individually. None of the individual antibiotics had an effect on the level of trophozoites present in the small intestine (Supplementary Data, S2). These findings indicate the necessity for the complete cocktail of antibiotics to alter the course of a *G. muris* infection.

**Figure 2:**
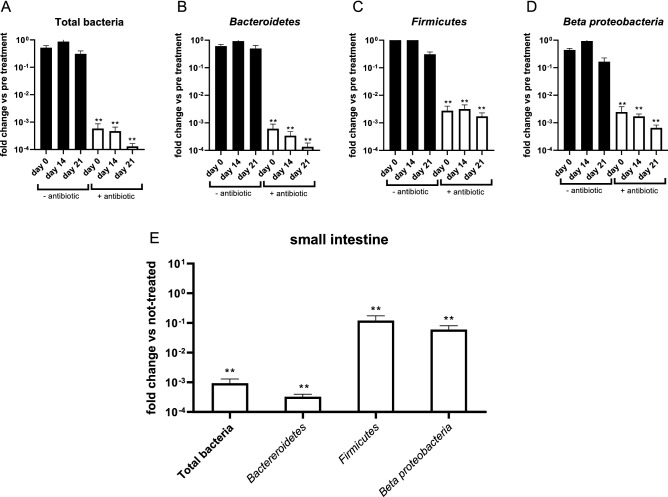
Effect of antibiotic treatment on the bacterial load in mice determined by qPCR on DNA extract of fecal (**A**–**D**) pellets and intestinal content (**E**). Results are shown for Total Bacteria, *Bacteroidetes*, *Firmicutes* and *Beta proteobacteria* as the mean of 5 mice with SEM errors bars. (**p ≤ 0.01).

We subsequently wished to determine the effectiveness of the antibiotic treatment by analysing both the total bacterial load as well as some of the more prevalent bacterial phyla typically present in mice (*Bacteroidetes*, *Firmicutes* and *Beta* proteobacteri*a*) by 16S rRNA qPCR. As shown in Fig. 2, the antibiotic treatment significantly reduced the total bacterial load present in the feces throughout the whole study (Fig. 2A). A similar effect was also measurable for the *Bacteriodetes*, *Firmicutes* and *Beta* proteobacteria (Fig. 2B–D). In the small intestine, the bacterial load was also strongly reduced, at least at the level of the total bacterial load and the phylum *Bacteroides.* The decrease for the *Firmicutes* and *Beta proteobacteria* on the other hand was smaller. Overall, these results show that the antibiotic treatment resulted in a strong reduction of the bacterial load in the digestive tract, yet the treated mice cannot be considered as ‘germfree’.

### The gut microbiome triggers the intestinal expression of antimicrobial peptides

Multiple studies have shown in the past that the presence of an intact intestinal microbiome contributes to an adequate innate immune response in the gut^20^. The interaction between the innate immune response and the intestinal microbiome is not only important for the homeostasis of the intestinal environment but also prevents intestinal colonisation of potential pathogenic infections. Therefore, an antibiotic-mediated depletion of the intestinal microbiome could potentially lead to an impaired innate immune barrier and subsequently enable the establishment of for example *G. duodenalis* in mice.

**Figure 3:**
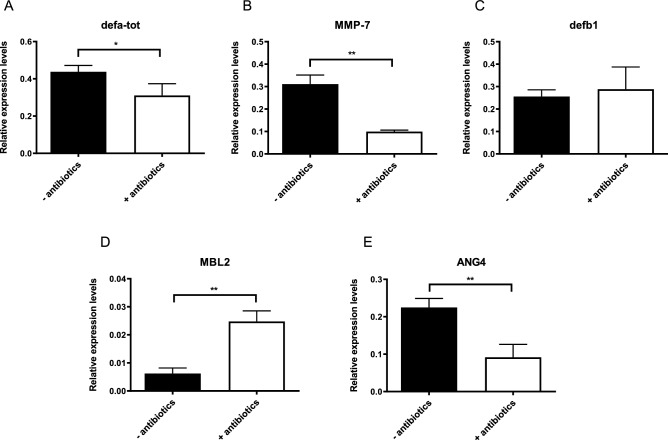
Effect of antibiotic treatment on the transcription of anti-microbial peptides and MBL2 in non-infected control mice without (black bar) and with (white bar) after 5 days of antibiotic cocktail treatment (**A**) α-defensins (defa-tot), (**B**) MMP-7, (**C**) defb1, (**D**) MBL2 and (**E**) ANG4. Relative mRNA expression levels were measured by qPCR. Mean transcription level from 5 mice are shown with SEM as error bars. (*p ≤ 0.05, **p ≤ 0.01).

One of the factors of the innate immune system that is important in the defense against a *Giardia* infection is the production of antimicrobial peptides (AMPs). These small peptides are mainly produced by Paneth cells and are excreted into the intestinal lumen where they are part of the complex mucosal barrier protecting the intestinal epithelial cell layer^21^. AMPs were previously shown to exhibit a toxic effect on *G. duodenalis* trophozoites in vitro^22^. In addition, transcriptional upregulation of α-defensins and β-defensin 1 (defb1) has been observed 21 days following a *G. muris* infection^7^, further indicating their importance. The influence of the microbiome on defensins has been suggested in a previous study as germfree mice displayed strongly reduced expression levels in the small intestine^23,24^. Therefore, a qPCR analysis in non-infected control mice was performed to compare expression levels of both total α-defensins (defa-tot) and defb1 between antibiotic treated mice and untreated mice. The results show that for defb1 (Fig. 3C) no effect was visible, while the mRNA expression levels of total α-defensins (Fig. 3A) on the other hand displayed a significant downregulation in antibiotic treated mice. After expression, α-defensins must be proteolytically cleaved by matrix metalloprotease-7 (MMP-7), as it contains an inhibitory amino-terminal pro-region^24^. It has been shown that mice deficient for MMP-7 show higher *G. duodenalis* trophozoite counts at day 13 p.i. compared to wild type mice, whilst there was no effect during a *G. muris* infection^25^. The importance of the intestinal microbiome on the expression levels of MMP-7 has already been studied in the past using germfree mice which showed undetectable levels of MMP-7 by immunohistochemistry^26^. Similarly, in this study we show that antibiotic mediated depletion of the intestinal microbiome results in a significant downregulation of MMP-7 (Fig. 3B). Yet, the biological relevance of this observation remains unclear as Ayabe et al.^27^ showed that basal MMP-7 levels in germ-free mice are already sufficient for the proteolytical cleavage of α-defensins. However, this study did not take into account what the effect would be when faced with a potential pathogen.

Other components of the innate immune system that are upregulated following a *Giardia* infection are the mannose binding lectin 2 (MBL2) and angiogenin 4 (ANG4). MBL2 is a circulating C-type lectin that is able to bind carbohydrate structures on pathogens in order to subsequently activate the complement cascade^28^. Previous studies have shown the importance of MBL2 during a *Giardia* infection as mice deficient for MBL2 displayed a delayed clearance of both *G. duodenalis*^29^ and *G. muris*^7^ compared to wild type mice. However, depletion of the intestinal microbiome actually resulted in an upregulation of MBL2 instead of a downregulation, indicating that the microbiome is not necessary for its expression (Fig. 3D). ANG4 was originally identified as a tumour-derived protein containing angiogenic properties. However, more recently it was also identified as an antimicrobial peptide, produced by Paneth cells, with bactericidal activity against both gram-negative as gram-positive bacteria^30^. Interestingly, ANG4 is one of the most upregulated genes following a *G. muris* infection^7^, yet its role during a *Giardia* infection still remains unclear. Just like for the other factors of the innate immune response, the expression levels of ANG4 were determined by qPCR between antibiotic treated mice and mice with a conventional microbiome. The results show a significant downregulation of ANG4 due to the absence of an intestinal microbiome resulting in a lower basal level of expression (Fig. 3E). In summary, these results indicate that the presence of several factors of the innate immune response in the intestinal lumen is decreased following antibiotic treatment, which could explain the increased susceptibility of antibiotic treated mice for a *G. duodenalis* infection.

### The intestinal microbiome is not essential for the initiation of the IL-17A response but stimulates IgA production and secretion

**Figure 4:**
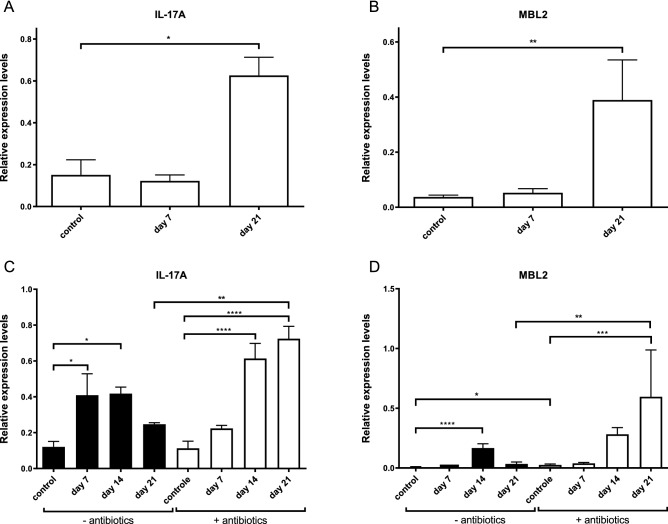
Kinetics of the intestinal IL-17A and MBL2 response following a *G. duodenalis* (**A**, **B**) and *G. muris* (**C**, **D**) infection in C75Bl/6 mice without (black bar) and with (white bar) antibiotic cocktail treatment. Relative mRNA expression levels were measured by qPCR at indicated time points. Mean transcription level from 5 mice are shown with SEM as error bars. (*p ≤ 0.05, **p ≤ 0.01, ***p ≤ 0.001, ****p ≤ 0.0001).

In normal conditions, *G. muris* infected mice develop a protective immune response in which upregulation of IL-17A is necessary for the elimination of the parasite^5^. Antibiotic treated mice however experience difficulties clearing a *G. muris* infection, suggesting an impaired IL-17 response. To determine the role of the intestinal microbiome on IL-17A induction, the expression levels of both IL-17A and its downstream effector MBL2 were measured by RT-qPCR in both antibiotic-treated and untreated mice following a *G. duodenalis* or *G. muris* infection. Previous reports have already shown an upregulation for both IL-17A and MBL2 within the first 3 weeks of a *G. muris* infection^5,7^. Consistent herewith, Fig. 4A and B demonstrate a significant upregulation of both IL17A and MBL2 at day 21p.i. The upregulation of IL-17A and MBL2 was also observed in the *G. muris* infected mice treated with the antibiotic cocktail (Fig. 4C,D) and was even stronger, most likely due to the higher parasitic load, in the antibiotic treated mice compared to the untreated mice. These observations indicate that the intestinal microbiome does not influence the induction process itself of the IL-17A response; yet, other downstream effectors of IL-17A in the immune response could still be affected by the microbiome.

**Figure 5:**
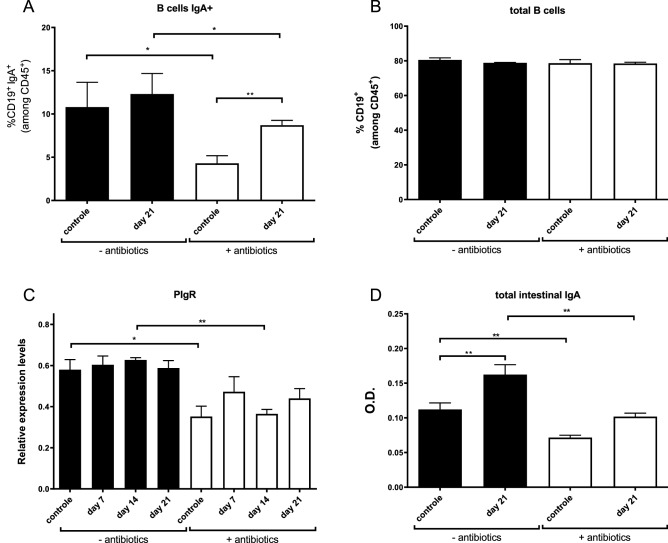
Effect of antibiotic treatment and *G. muris* infection on the intestinal production and secretion of IgA. (**A**) Relative percentage of IgA producing B-cells within the CD45^+^ cell population isolated from the Peyer’s patch tissue. (**B**) Relative percentage of total B-cells within the CD45^+^ cell population isolated from the Peyer’s patch tissue. (**C**) Relative mRNA expression levels of PIgR measured by qPCR at indicated time points. (**D**) Total intestinal IgA levels. Results are shown as the mean from 5 mice with SEM as error bars. (*p ≤ 0.05, **p ≤ 0.01).

One of the most important downstream effectors of IL-17A following a *Giardia* infection is the stimulation of IgA production by B-cells. The necessity of B-cell development and subsequent IgA production in the protection against *Giardia* was contested at first, as several studies had contradicting outcomes^31,32^, yet studies performed in IgA deficient mice have shown a strongly delayed clearance of both *G. muris* as *G. duodenalis*^6^. To investigate the influence of the microbiome on IgA levels in the intestinal lumen, several approaches were used in this study. In a first step, flow cytometry was performed to analyse the distribution of several B-cell types in the Peyer’s patches of the small intestine. Figure 5B shows that microbiome depletion did not result in altered amounts of total B-cells, yet, the amount of IgA-producing B-cells was significantly decreased in the antibiotic treated mice, both in control and infected mice (Fig. 5A). Besides IgA-expressing B-cells, also IgM positive B-cells were decreased due to the antibiotic treatment but only between the control groups (Supplementary Data S3), indicating a compensation of other B-cell subpopulations for the decrease in IgA^+^ and IgM^+^ while total B-cells remained equal. Second, the expression levels of the polymeric immunoglobulin A receptor (PIgR) were examined by qRT-PCR. PIgR is an essential transporter for IgA in order to translocate from the lamina propria, where it is produced by B-cells, to the intestinal lumen^33^. Previous studies have shown that also PIgR is partially regulated by an IL-17 induction^34^. As observed in Fig. 5C, intestinal microbiome depletion led to a significant downregulation of PIgR in control mice at day 14 p.i. and hinted to a downward trend in infected mice. Collectively, the above findings suggest that antibiotic-mediated depletion of the microbiome causes a decrease in luminal IgA as a result of decreased numbers of IgA^+^ B-cells in the Peyer’s patches concomitant with IgA transporter downregulation. In order to confirm this hypothesis, total IgA levels in the intestinal lumen were measured by an IgA ELISA on luminal extracts isolated at day 21 p.i. (Fig. 5D). As described in previous studies, the amount of luminal IgA increases in the mice both with and without antibiotics when infected with *G. muris*. However, intestinal microbiome depletion results in lower IgA levels in control as well as in infected mice, which could explain the reduced clearance of a *G. muris* infection following antibiotic treatment.

### Gut microbiota influences the intestinal motility

An often forgotten part of the intestinal immune response is the expulsion of invading pathogens by the infected host through increased intestinal motility. This process is a complex regulated system depending on multiple factors. Studies in the past have shown that several intestinal pathogenic infections, including *Giardia*, led to an increased intestinal motility, in which the host attempts to clear the infection rapidly^35,36^.

**Figure 6:**
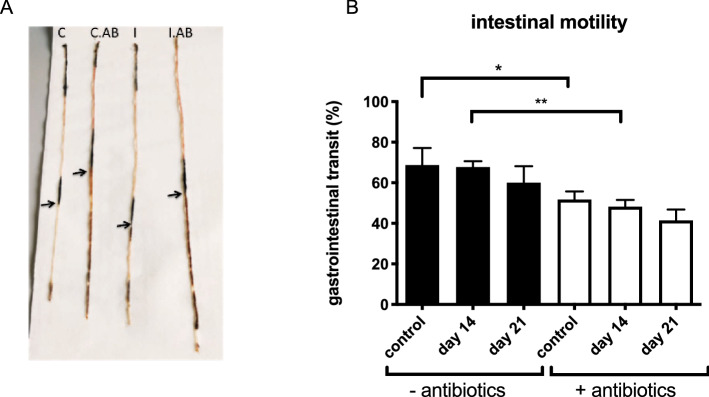
Effect of antibiotic treatment and *G. muris* infection on the intestinal motility. (**A**) Picture of murine small intestine, 20 min after oral administration of 10% charcoal solution. (**B**) Kinetics of intestinal transit following a *G. muris* infection in C75 BL/6 mice without (black bar) and with (white bar) antibiotic treatment. Results are shown as the mean from 5 mice with SEM as error bars. (*p ≤ 0.05, **p ≤ 0.01).

As stated above, gut microbiome depletion induces severe physiological changes of the digestive tract. These findings led to the consideration that depletion possibly alters the intestinal motility as well. To test this hypothesis, intestinal motility was determined by the distance an orally administrated dye travelled through the digestive tract following infection with *G. muris* (Fig. 6A). Unlike previous studies, our results do not show increased intestinal motility upon a *G. muris* infection. Yet, gut microbiome depletion by an antibiotic treatment did impact intestinal motility. Both in the non-infected controls as in the *G. muris* infected animals, treatment led a longer intestinal transit time (Fig. 6B). As a consequence of decreased intestinal motility, *Giardia* trophozoites are more slowly expulsed from the small intestine and subsequently may reside longer in the intestinal lumen. Together with a compromised immune response (as indicated by the results described above), these findings may allow explaining the prolonged phenotype of a *G. muris* infection and the increased susceptibility of antibiotic-treated mice to a *G. duodenalis* infection.

## Conclusion

The outcome of this study clearly highlights the importance of the intestinal microbiome in the *Giardia*-mice immune interaction. Not only did the *G. muris* infection in microbiome-depleted mice show a more chronic course over time, it also resulted in a higher parasite burden. Surprisingly, the IL-17A induction mediated by *Giardia* was still intact suggesting that IL-17A upregulation occurs independently of an intact intestinal microbiome. Nonetheless, several immune effectors mechanisms were compromised in the absence of the intestinal microbiota. Those factors belonged to both innate (AMPs and intestinal transit) and adaptive (IgA) immune responses. Our findings show that IL-17A induction on its own is not sufficient for clearance of a *G. muris* infection but additionally requires priming of the immune system by the intestinal microbiome. Furthermore, the decrease of components important in the innate immune system, such as the defensins, Ang4 and intestinal motility, could potentially explain the increased susceptibility of microbiome-depleted mice to a *G. duodenalis* infection.

In addition to the new insights into the interaction between the parasite, the microbiome and the host immune system, the outcome of this study also highlights the potential danger of antibiotic usage during a *Giardia* infection. Giardiasis is often misdiagnosed at first, resulting in the prescription of antibiotics which are ineffective against *Giardia*^37^. Even apart from the issues concerning antibiotic resistance, the results presented here show that mistreatment of *Giardia* with antibiotics could result in a more severe and prolonged infection. Importantly however, the duration of such an effect, after the termination of the antibiotic treatment, is currently not known and requires further research.

## Supplementary Information

Supplementary Information.
