# Supplementary material for: Regulatory role of the intestinal microbiota in the immune response against Giardia
Source: Sci Rep. 2021 May 19;11:10601. doi: 10.1038/s41598-021-90261-z (PMC8134572; doi:10.1038/s41598-021-90261-z)

Regulatory role of the intestinal microbiota in the immune response against *Giardia*.

Maertens B[1], Gagnaire A[1], Paerewijck O[1], De Bosscher K[2], Geldhof P*[1]

[1]Department of Virology, Parasitology and Immunology, Laboratory of Parasitology, Faculty of Veterinary Medicine, Ghent University, Merelbeke, Belgium, Peter.Geldhof@Ugent.be

[2]VIB Department of Medical Protein Research, Translational Nuclear Receptor Research lab, Faculty of Medicine and Health Sciences, Ghent University, Ghent, Belgium

*Corresponding author: peter.geldhof@ugent.be

Supplementary table S1: Primer sequences used for the quantitative PCR analyses.

Gene	Primer sequence
mTBP	F: CAAACCCAGAATTGTTCTCCTT<br>R: ATGTGGTCTTCCTGAATCCCT
mHPRT1	F: TGGATACAGGCCAGACTTTGTT<br>R: CAGATTCAACTTGCGCTCATC
mIL-17A	F: TGACAGTGGTTTATGCAGAGAC<br>R: CGTCACGTCCATCTTTGCC
mMBL2	F: ACTCCCTGAAGAATATACCCTCC<br>R: CGCTATTGAGCACAGATACGAG
mdefa-tot	F: GGTGATCATCAGACCCCAGCATCAGT<br>R: AAGAGACTAAAACTGAGGAGCAGC
mdefb1	F: GAGCGGAGACAGAATCCTCC<br>R: TCTTTTCTCCCAGATGGAGC
mMMP-7	F: GCATTTCCTTGAGGTTGTCC<br>R: CACATCAGTGGGAACAGGC
mAng4	F: GGTTGTGATTCCTCCAACTCTG<br>R: CTGAAGTTTTCTCCATAAGGGCT
mPIgR	F: ATGAGGCTCTACTTGTTCACGC<br>R: CGCCTTCTATACTACTCACCTCC
total bacteria (27F_a & 338R_a)	F: AGAGTTTGATYMTGGCTCAG<br>R: GCTGCCTCCCGTAGGAGT
Bacteroidetes (Bac960F & Bac1100R)	F: GTTTAATTCGATGATACGCGAG<br>R: TTAASCCGACACCTCACGG
Firmicutes (Firm934F & Firm1060R)	F: GGAGYATGTGGTTTAATTCGAAGCA<br>R: AGCTGACGACAACCATGCAC
Betaproteobacteria (Beta979F & Beta1130R)	F: AACGCGAAAAACCTTACCTACC<br>R: TGCCCTTTCGTAGCAACTAGTG

Supplementary figure S1: Visible effect on the cecum in antibiotic-treated mice. The cecum is indicated with an arrow.

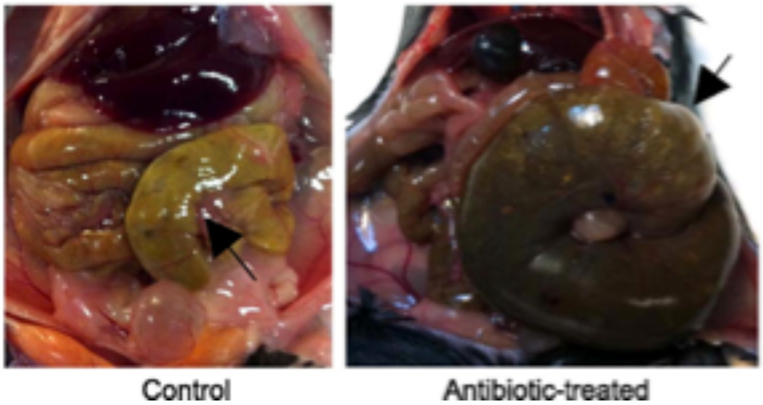

Supplementary figure S2: Effect of the individual antibiotics neomycin, ampicillin and vancomycin on *G. muris* trophozoite counts at day 21 p.i..

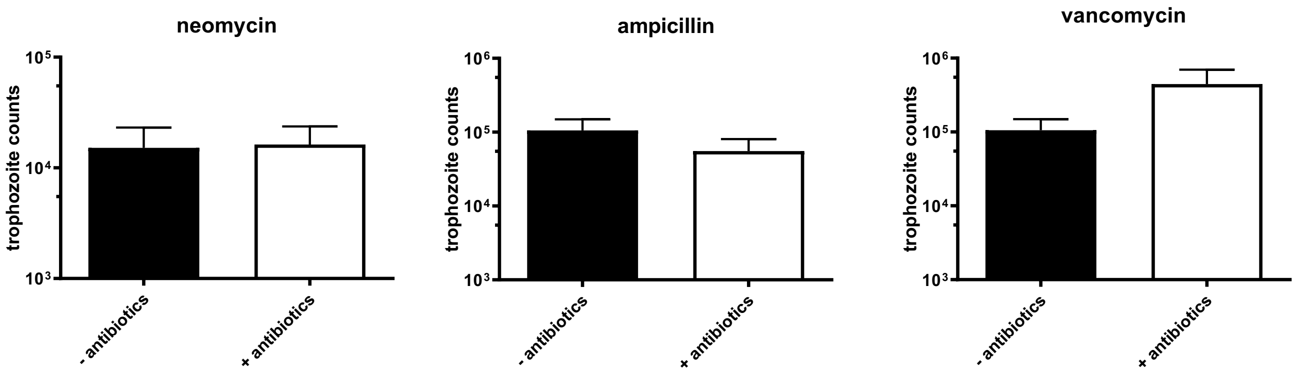

Supplementary figure S3: Effect of antibiotic treatment on the cellular populations in Peyer's patch tissue.

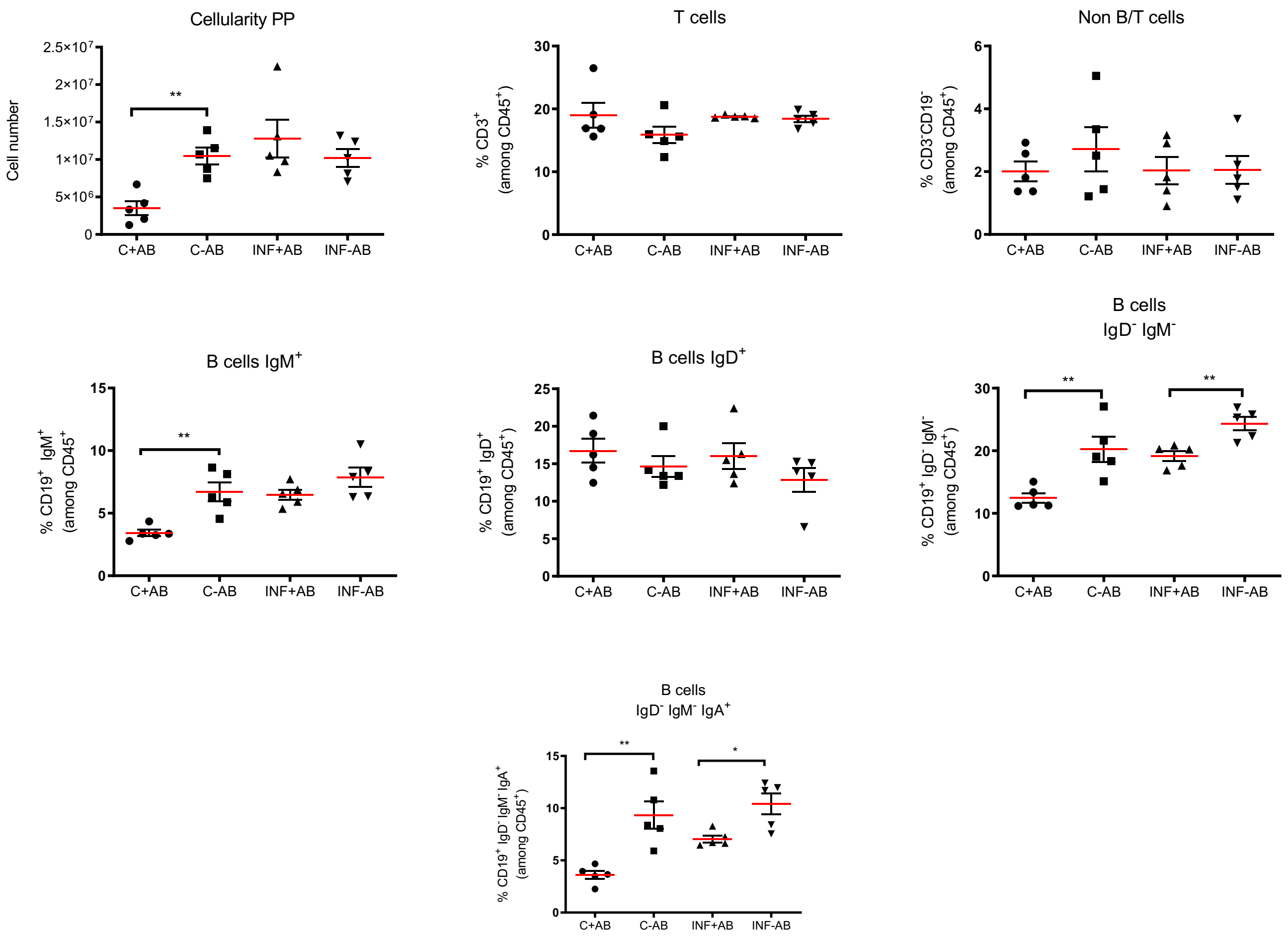

Supplement: Supplementary file 1 — Supplementary Information. [file 41598_2021_90261_MOESM1_ESM.pdf]
